# Small Bowel Adenocarcinoma Arising in Long-Standing Stricturing Crohn’s Disease: Diagnostic Challenges and Therapeutic Dilemmas

**DOI:** 10.7759/cureus.108633

**Published:** 2026-05-11

**Authors:** Salma Mechhor, Manal Cherkaoui, Hicham El Bacha, Oumeima Cherkaoui Malki, Nadia Benzzoubeir, Ikram Errabih

**Affiliations:** 1 Medicine B Unit, Ibn Sina Teaching Hospital - Mohamed V University, Rabat, MAR

**Keywords:** crohn´s disease, fistula, immunosuppressive therapy, mucinous adenocarcinoma, small bowel adenocarcinoma

## Abstract

Small bowel adenocarcinoma (SBA) is a rare but severe complication of long-standing Crohn’s disease (CD), particularly in patients with stricturing phenotypes. Its diagnosis is often delayed due to overlapping clinical and radiological features with inflammatory disease.

We report the case of a 52-year-old man with an 18-year history of stricturing and fistulizing ileal CD, previously treated with azathioprine and adalimumab. Despite clinical remission, magnetic resonance enterography performed during therapeutic reassessment revealed complex stricturing disease with fistulizing features. Surgical resection was undertaken. Histopathological examination revealed mucinous adenocarcinoma with a signet-ring cell component exceeding 30%, staged as pT2N0 after complete (R0) resection.

Postoperative management included close radiological surveillance without adjuvant chemotherapy. Given the recent cancer diagnosis, a temporary treatment-free strategy for CD was initially adopted, with monitoring of fecal calprotectin levels. Endoscopic recurrence led to the introduction of ustekinumab. At 12-month follow-up, the patient remains clinically stable with no evidence of oncological recurrence.

This case highlights the diagnostic challenge of SBA in CD, particularly in clinically quiescent patients. It underscores the absence of validated screening strategies and emphasizes the importance of multidisciplinary decision-making in balancing oncologic safety and inflammatory disease control.

## Introduction

Complications of Crohn’s disease (CD) include small bowel adenocarcinoma (SBA), one of the most serious due to its rarity and poor prognosis. Its incidence in patients with CD is significantly higher than in the general population, estimated at 8.3 per 100,000 person-years [1]. Reported risk factors include long disease duration, male gender, distal small bowel involvement, and use of immunosuppressive therapy. A particular challenge in this population is that the clinical presentation often overlaps with fibrostenotic CD, including obstruction, weight loss, and abdominal pain, thereby delaying diagnosis and worsening prognosis [2]. This highlights the dual role of surgery as both a diagnostic and therapeutic intervention.

## Case presentation

We report the case of a 52-year-old man with an 18-year history of stricturing and fistulizing small-bowel CD. Twelve years prior, he had undergone ileal resection for subocclusive symptoms related to terminal ileal stenosis. He was subsequently treated with azathioprine, followed by adalimumab for four years.

**Figure 1:**
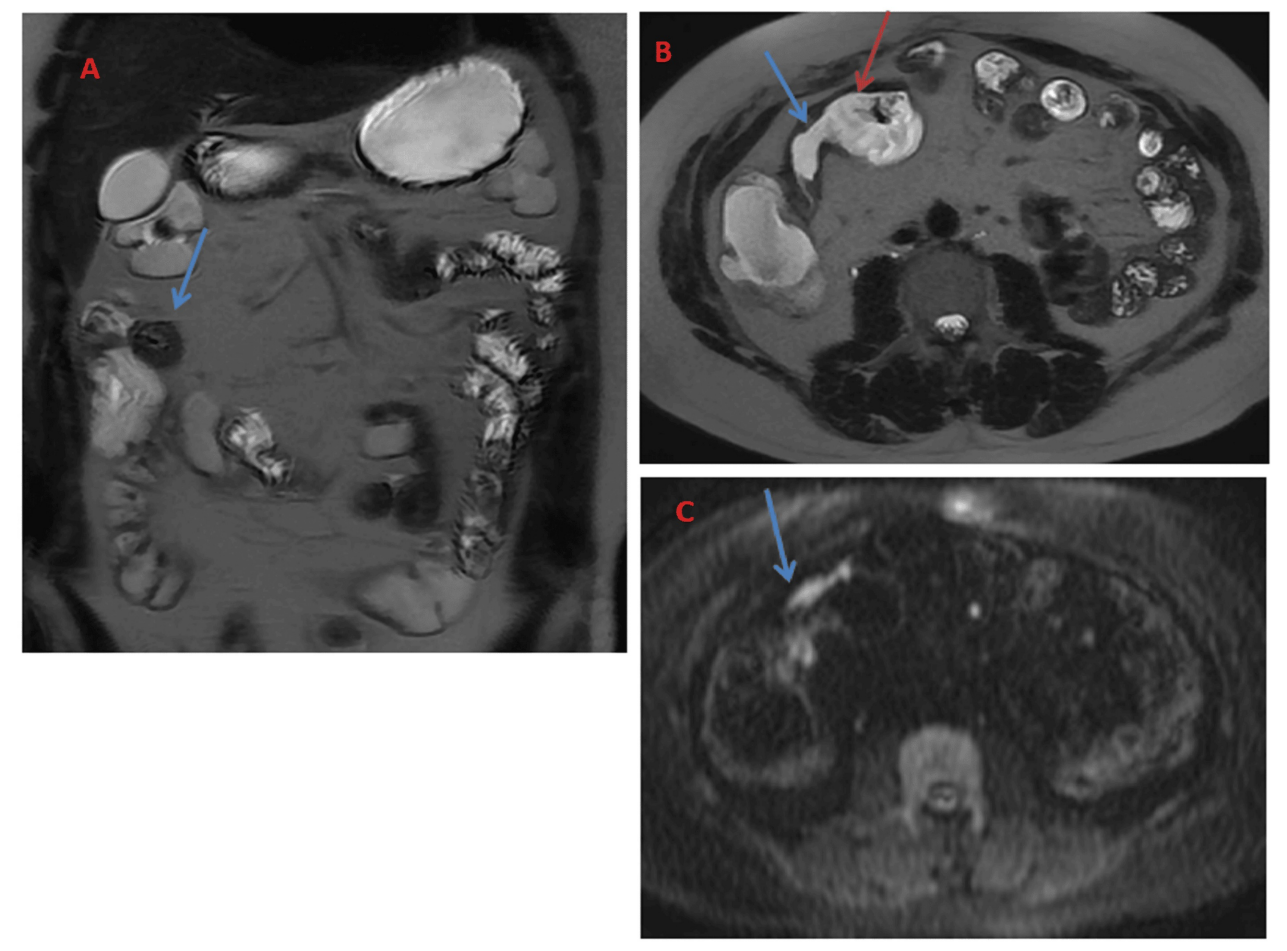
Magnetic resonance enterography showing terminal ileal wall thickening and stricturing disease. (A) Coronal T2-weighted MRI enterography showing asymmetric circumferential wall thickening of the terminal ileum. (B) Axial T2-weighted MRI enterography showing irregular stenosing wall thickening of the terminal ileum with digestive distension of the upstream loop. (C) Diffusion-weighted MRI enterography showing irregular wall thickening of the terminal ileum with hyperintense signal on diffusion.

Being clinically asymptomatic, with a CDAI score of 70 and a preserved quality of life (IBDQ score 265/280), reassessment was performed in the context of a potential therapeutic de-escalation strategy. Fecal calprotectin was 210 μg/g. Magnetic resonance enterography demonstrated a 5-cm segment of asymmetric terminal ileal wall thickening (maximum thickness 13 mm) with 6 cm upstream dilatation. A second short stenotic segment located 5 cm proximally reduced the lumen to 8 mm over 15 mm and was associated with an entero-mesenteric fistula (Figure 1).

**Figure 2:**
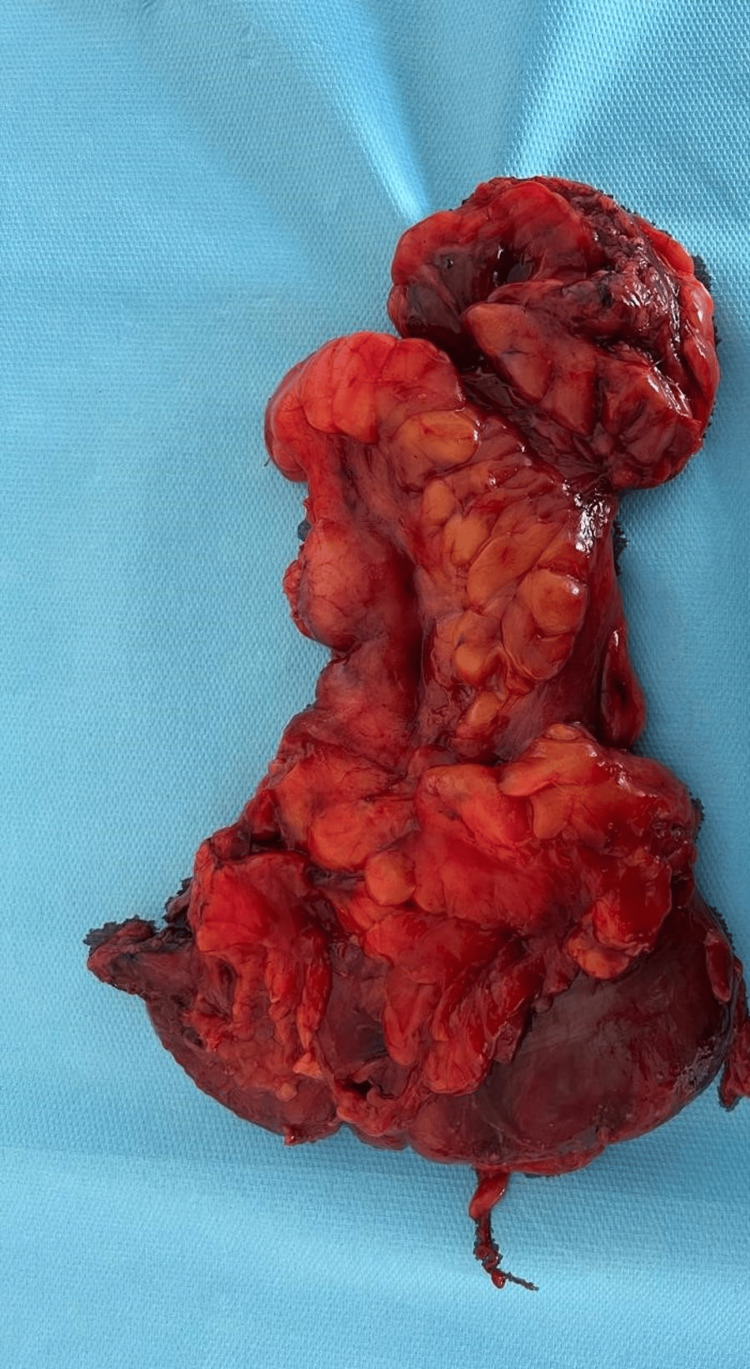
Surgical finding. Image of the resected ileal loop specimen.

Following multidisciplinary discussion, surgical management was undertaken given the stricturing nature of the bowel wall thickening and the presence of a fistula. Intraoperative findings included dense adhesions, a stenosis 20 cm proximal to the ileocecal valve with mild upstream dilatation and an entero-enteric fistula, an entero-mesenteric fistula within the pelvis, and a second stricture at the ileocecal valve. Adhesiolysis, fistula division, and segmental ileal resection, including the stenotic and fistulizing segments, were performed, followed by side-to-side ileocolic anastomosis (Figure 2).

**Figure 3:**
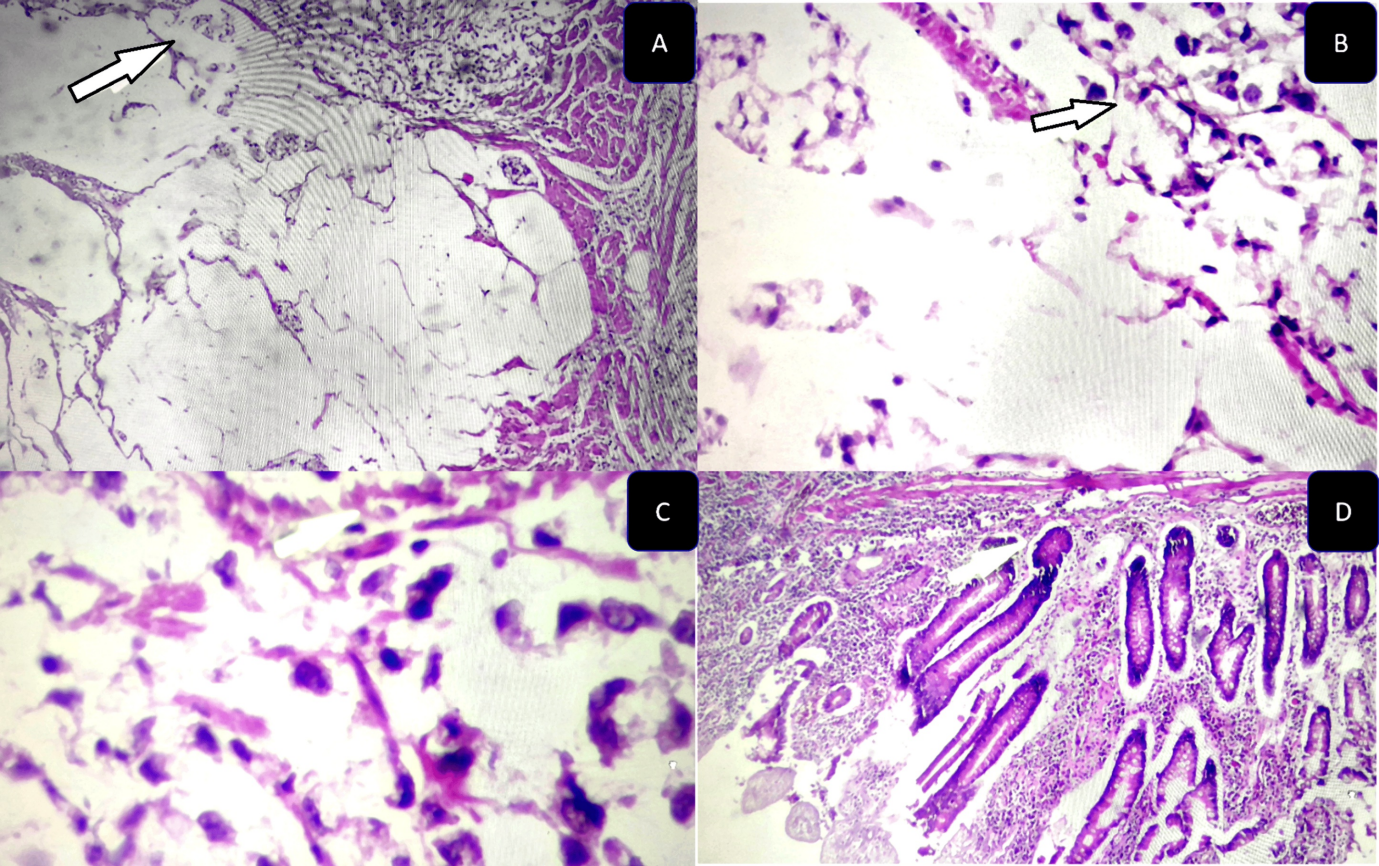
Histological findings. Histological findings of mucinous adenocarcinoma (A to C), showing neoplastic cells embedded in an abundant extracellular mucin, A low power, B medium power, and C High power. D: adjacent mucosa showing architectural distortion and abundant inflammatory infiltrate consistent with Crohn's disease.

Histopathological examination revealed mucinous adenocarcinoma with a 30% signet-ring cell component infiltrating the muscularis propria: tumor size was 2 cm, with evidence of vascular emboli and no perineural invasion. Surgical resection margins were free of tumor, measuring 4 cm proximally and 14 cm distally. Pathological staging was pT2N0 according to the American Joint Committee on Cancer 8th edition (2017) [3] classification (Figure 3).

The case was reviewed in an oncologic multidisciplinary meeting. Given the absence of nodal involvement, early-stage disease (stage I), and complete R0 resection, adjuvant chemotherapy was not indicated. A strategy of radiologic surveillance with abdominal CT every three months was adopted.

Considering the recent cancer diagnosis, a temporary treatment-free interval for CD was initially pursued, with close monitoring using serial fecal calprotectin measurements. A progressive rise to 542 μg/g prompted ileocolonoscopy, which demonstrated ulcerated neo-terminal ileitis without macroscopic or microscopic evidence of tumor recurrence.

After reassessment in an inflammatory bowel disease multidisciplinary meeting, and in light of prior thiopurine and anti-TNF exposure, ustekinumab was initiated. At 12-month follow-up, the patient remains clinically stable under ustekinumab, without radiologic evidence of oncologic recurrence. Currently, screening includes abdominal CT every six months. The most recent fecal calprotectin level was 220 μg/g.

## Discussion

SBA is a rare but well-recognized complication of long-standing CD, first described in 1956. Despite decades of reports, it remains an uncommon entity, with an estimated absolute risk of 2.4 per 10,000 patient-years in CD [1]. However, population-based studies have consistently shown a markedly increased relative risk compared with the general population, ranging from 6- to >60-fold [2,4].

SBA in CD predominantly arises in the terminal ileum, typically after prolonged disease duration and in patients with stricturing or fistulizing phenotypes [5]. Identified risk factors include long-standing disease, early age at onset, prior small bowel resection, penetrating behavior, and smoking [1,5]. Our patient presented several of these risk features, notably 19 years of ileal disease and a penetrating phenotype.

One of the major clinical challenges is that SBA frequently mimics active CD. Symptoms such as abdominal pain, subocclusion, or imaging-detected strictures are often attributed to inflammatory or fibrotic disease rather than malignancy [6]. Consequently, diagnosis is commonly delayed and often made intraoperatively, with most reported cases being stage T3 or T4 at diagnosis, frequently with nodal involvement (Table 1) [5,7]. In contrast, our patient was diagnosed at a relatively early pathological stage (pT2N0 on pathological findings), despite vascular emboli, which is uncommon in the literature.

**Table 1:** Reported cases and series of small bowel adenocarcinoma associated with Crohn’s disease.

Author	Year	Journal	N cases	Mean Age	Location	Stage at Diagnosis	Notes
Cahill et al. [5]	2014	World J Gastro-enterol	Review (220 cases)	~50 yrs	Terminal ileum	Mostly T3/T4	Frequent nodal involvement
Benesch et al. [6]	2023	World J Oncol	Systematic review (SRCC)	~59 yrs	Distal ileum	Advanced	High proportion SRCC
Kothadia et al. [4]	2019	Case Rep Oncol Med	1	31 yrs	Ileum	T4N1M1	Metastatic, prolonged survival

This case is particularly illustrative, as the patient was clinically quiescent at the time of therapeutic reassessment, with CDAI-compatible remission, preserved quality of life, and no obstructive symptoms. The decision to proceed to surgery was based on radiological evidence of disease activity, in the absence of features suggestive of malignant transformation, such as irregular wall thickening, mass formation, lymphadenopathy, or exophytic lesions. Despite these reassuring findings, malignancy was only identified postoperatively, highlighting a major diagnostic pitfall in long-standing stricturing CD. This case underscores that SBA may develop silently and remain undetected in clinically quiescent disease. Currently, no standardized screening strategies exist for early detection of SBA in this population [8]. The systematic review by Uchino et al. confirmed the absence of consensus or validated surveillance protocols [8], and ECCO guidelines similarly do not recommend routine screening due to the rarity of the condition and the lack of cost-effective tools [9].

Histologically, our case is notable for mucinous adenocarcinoma with a 30% signet-ring cell component. Recent literature suggests that signet-ring cell adenocarcinoma (SRCC) may be overrepresented in CD-associated SBA and may occur at younger ages and with more aggressive behavior [7]. A recent review reported that nearly all cases were diagnosed at advanced T stages, reinforcing the aggressive biological profile of this subtype [7]. Although our case was not predominantly SRCC, the presence of this component supports the hypothesis of inflammation-driven carcinogenesis with mucinous differentiation.

Another critical issue raised by this case concerns the potential role of anti-TNF therapy in carcinogenesis. Long-term exposure to adalimumab preceded the diagnosis. However, current evidence does not support an increased overall cancer risk with anti-TNF monotherapy in CD [10]. Large cohort studies and ECCO guidelines indicate no clear signal of increased malignancy risk attributable to anti-TNF agents alone [9,10]. Moreover, it remains difficult to disentangle the respective roles of chronic inflammation versus immunosuppression. Chronic transmural inflammation is a well-established carcinogenic driver in IBD [1,5], and it is more biologically plausible that persistent inflammatory activity, even if subclinical, plays a central role.

The management of CD following curative resection of SBA poses a therapeutic dilemma: balancing the risk of oncologic recurrence against the need for inflammatory control. ECCO guidelines emphasize that decisions must be individualized and multidisciplinary, taking into account cancer type, stage, and IBD severity [9]. Data on optimal timing of biologic reintroduction remain limited and heterogeneous [9,11]. Among available agents, ustekinumab appears to have one of the most reassuring safety profiles regarding malignancy recurrence. Observational data have not demonstrated increased rates of new or recurrent cancers in IBD patients treated with ustekinumab, including those with prior malignancy [11]. Although long-term data remain limited, the cumulative evidence is globally reassuring. Similarly, vedolizumab, a gut-selective anti-integrin agent, is considered a safe option in patients with prior malignancy, with observational data showing no increased risk of incident cancer compared to TNF-α antagonists [12]. However, in our setting, vedolizumab was not available, and ustekinumab was selected based on its reassuring safety profile and accessibility, in line with an individualized and multidisciplinary decision-making approach.

In our patient, rising fecal calprotectin levels and endoscopic recurrence justified therapeutic reintroduction. Given the curative (R0) surgery, pT2N0 staging, and absence of metastatic disease, ustekinumab was selected based on its favorable oncologic safety profile and ECCO-supported individualized decision-making framework [9,11].

## Conclusions

This case highlights several important clinical messages. First, SBA must be considered in long-standing ileal CD with stricturing behavior, even in the absence of overt symptoms. Second, no validated screening strategy exists, reinforcing the importance of careful radiologic evaluation in complex disease. Third, current evidence does not implicate anti-TNF therapy as a primary carcinogenic driver. Finally, post-cancer IBD management requires nuanced, multidisciplinary decision-making, where newer biologics such as ustekinumab may offer a reasonable balance between inflammatory control and oncologic safety.
